# New O-Aryl-Carbamoyl-Oxymino-Fluorene Derivatives with MI-Crobicidal and Antibiofilm Activity Enhanced by Combination with Iron Oxide Nanoparticles

**DOI:** 10.3390/molecules26103002

**Published:** 2021-05-18

**Authors:** Ilinca Margareta Vlad, Diana Camelia Nuță, Robert Viorel Ancuceanu, Miron Teodor Caproiou, Florea Dumitrascu, Ioana Cristina Marinas, Mariana Carmen Chifiriuc, Luminita Gabriela Măruţescu, Irina Zarafu, Ioana Raluca Papacocea, Bogdan Ștefan Vasile, Adrian Ionuț Nicoară, Cornelia-Ioana Ilie, Anton Ficai, Carmen Limban

**Affiliations:** 1 Department of Pharmaceutical Chemistry, Faculty of Pharmacy, “Carol Davila” University of Medicine and Pharmacy, 6 Traian Vuia, 020956 Bucharest, Romania; ilinca.vlad@drd.umfcd.ro (I.M.V.); diana.nuta@umfcd.ro (D.C.N.); carmen.limban@umfcd.ro (C.L.); 2 Department of Pharmaceutical Botany, Faculty of Pharmacy, “Carol Davila” University of Medicine and Pharmacy, 6 Traian Vuia, 020956 Bucharest, Romania; robert.ancuceanu@umfcd.ro; 3 Costin D. Nenițescu” The Organic Chemistry Centre of Romanian Academy, 202B Splaiul Independenței, 060023 Bucharest, Romania; dorucaproiu@gmail.com (M.T.C.); fdumitra@yahoo.com (F.D.); 4 Department of Microbiology, Faculty of Biology & Research Institute of the University of Bucharest (ICUB), University of Bucharest, 060101 Bucharest, Romania; ioana.cristina.marinas@gmail.com (I.C.M.); carmen.chifiriuc@bio.unibuc.ro (M.C.C.); 5 Academy of Romanian Scientists, 3 Ilfov Street, 050045 Bucharest, Romania; 6 Department of Organic Chemistry, Biochemistry and Catalysis, Faculty of Chemistry, University of Bucharest, 4-12 Regina Elisabeta, 030018 Bucharest, Romania; zarafuirina@yahoo.fr; 7 Department of Physiology I, Faculty of Medicine, “Carol Davila” University of Medicine and Pharmacy, 8 Eroilor Sanitari, 050474 Bucharest, Romania; rpapacocea@gmail.com; 8 Faculty of Applied Chemistry and Materials Science, University POLITEHNICA of Bucharest, 313 Spl. Independentei, 060042 Bucharest, Romania; bogdan.vasile@upb.ro (B.Ș.V.); adrian.nicoara@upb.ro (A.I.N.); cornelia_ioana.ilie@upb.ro (C.-I.I.)

**Keywords:** 9-fluorenone, 9*H*-fluoren-9-one oxime, O-aryl-carbamoyl-oxymino-fluorene derivatives, antimicrobial, antibiofilm, iron oxide nanoparticles

## Abstract

Antimicrobial resistance is one of the major public health threats at the global level, urging the search for new antimicrobial molecules. The fluorene nucleus is a component of different bioactive compounds, exhibiting diverse pharmacological actions. The present work describes the synthesis, chemical structure elucidation, and bioactivity of new O-aryl-carbamoyl-oxymino-fluorene derivatives and the contribution of iron oxide nanoparticles to enhance the desired biological activity. The antimicrobial activity assessed against three bacterial and fungal strains, in suspension and biofilm growth state, using a quantitative assay, revealed that the nature of substituents on the aryl moiety are determinant for both the spectrum and intensity of the inhibitory effect. The electron-withdrawing inductive effect of chlorine atoms enhanced the activity against planktonic and adhered *Staphylococcus aureus*, while the +I effect of the methyl group enhanced the anti-fungal activity against *Candida albicans* strain. The magnetite nanoparticles have substantially improved the antimicrobial activity of the new compounds against planktonic microorganisms. The obtained compounds, as well as the magnetic core@shell nanostructures loaded with these compounds have a promising potential for the development of novel antimicrobial strategies.

## 1. Introduction

Antimicrobial resistance represents one of the major threats to public health worldwide, causing 700,000 deaths annually, and the number is expected to rise to 10 million in 2050. The dramatic decrease in the effectiveness of the currently available treatments is urging the immediate discovery of novel antibiotics [1]. However, the interest of pharmaceutical industry seems to remain low in investing in the field of antibiotics [2], needing to be counterbalanced by a stronger commitment of the academic researchers for the development of novel antimicrobial strategies.

**Figure 1:**
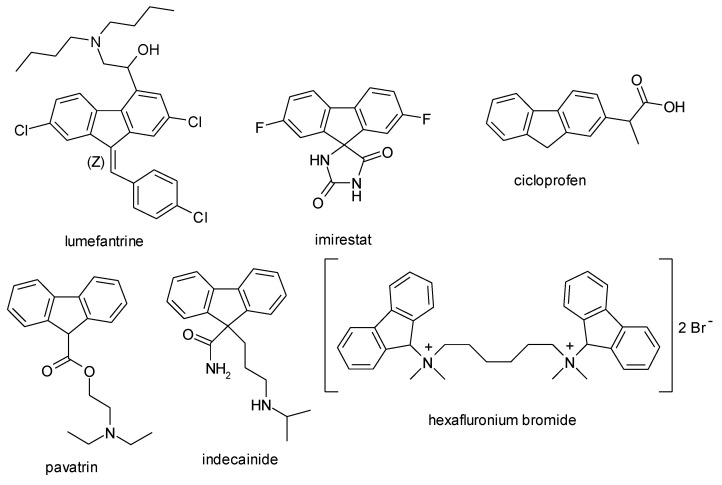
Fluorene—core drugs.

Fluorene, a tricyclic aromatic hydrocarbon, is found in the structure of some drugs, such as lumefantrine (antimalarial), imirestat (aldozreductase inhibitor), cicloprofen (analgesic, anti-inflammatory agent), pavatrin (antispasmodic), indecainide (antiarrhythmic), and hexafluronium bromide (muscle relaxant, a nicotinic acetylcholine receptor antagonist) (Figure 1).

A number of new fluorene derivatives were obtained using as the basic intermediate *N*-octadecyl-9-oxo-9*H*-fluoren-4-carboxamide. The synthesized derivatives react with propylene oxide, resulting in compounds with good surface-active properties. Therefore, these compounds can be used in different applications as drugs, wetting agents, moderate emulsifiers, household detergents, paints, and also in the cosmetic and textile industry [3]. The biological degradation of fluorene-based surfactants was more than 96% after seven days. Most of the compounds exhibited variable inhibitory effects on Gram-positive (*Bacillus subtilis*, *Staphylococcus aureus*) and Gram-negative (*Pseudomonas aeruginosa*, *Escherichia coli*) bacterial strains, as well as on fungal strains (*Aspergillus niger*, *Candida albicans* and *Curvularia* spp.).

2-(9*H*-fluorene-2-yl)-3-phenyl-bicyclo[2.2.1]hept-5-ene-2-yl methanone derivatives, which have the phenyl radical substituted by various atoms or functional groups, were obtained through the Diels-Alder synthesis and have shown in vitro antimicrobial and antioxidant properties [4].

Among the 2-(9*H*-fluoren-9-yl-methoxycarbonylamino)-propionic acid 3-(2-substituted-quinolin-3-yl)-isoxazol-5-yl-methyl esters, having the quinoline ring 2-substituted with various radicals, compounds with -SH and -SeH groups, exhibited good antibacterial activity against Gram-positive and Gram-negative bacteria (*Streptococcus pyogenes*, *Staphylococcus aureus*, *Pseudomonas aeruginosa* and *Bacillus subtillis*), compared to the ciprofloxacin standard [5].

**Figure 2:**
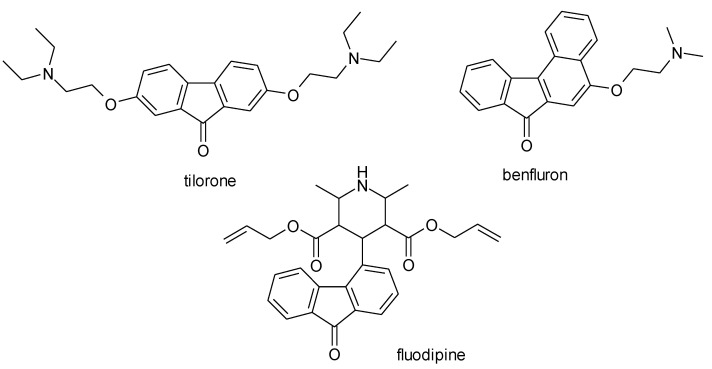
9-Fluorenone derivatives drugs.

Fluorenones have various therapeutic applications. Thus, tilorone has antiviral properties, benfluron is antineoplastic, and fluodipine is a cardiodepressant agent. The 9-fluorenone class includes many potentially active substances, which can be studied to optimize lead molecules in several therapeutic areas (Figure 2).

The study of the influence of tilorone (2,7-bis(2-diethylaminoethoxy)fluoren-9-one) on the activity of *Staphylococcus aureus* DnaG primase showed that it is inhibited by tilorone, although no inhibition of staphylococcal growth in the presence of this compound was observed. Structural modulations were performed to facilitate the diffusion of the compound into the bacterial cell, to inhibit bacterial growth and also to identify a promising lead compound. Thus, different derivatives of 9-fluorenone were synthesized, using tilorone as structural model, and adding hydrocarbon chains with different lengths and different terminal groups. These compounds were tested against Gram-positive and Gram-negative bacteria, and some exhibited a relatively low inhibitory concentration on *Bacillus anthracis*, methicillin-resistant *Staphylococcus aureus*, *Burkholderia thailandensis,* and *Francisella tularensis*. The results also demonstrated that the biodistribution of tilorone can be improved by further altering the chain length, the resulting compounds being used as prototype molecules to design and develop future stronger and more potent antimicrobial drugs [6].

In addition, 9-Fluorenon-4-carboxamides have been obtained as tilorone analogues and their cytotoxicity, antiviral, and cytokine-inducing properties have been demonstrated [7].

Using 9-fluorenone, Schiff bases were synthesized and investigated for their biological activity using molecular docking against a bacterial protein (*Proteus mirabilis* catalase). Among the tested molecules, N,N’-bis-Fluoren-9-yliden-ethane-1,2-diamine revealed the highest docking score (81,947). Some of the compounds exhibited antimicrobial activity against strains of *Escherichia coli*, *Staphylococcus aureus*, *Pseudomonas aeruginosa*, *Proteus mirabilis* and *Klebsiella pneumoniae* comparable to the standard antibiotic, streptomycin, at a concentration of 100 μg/mL [8].

A new series of 2,7-diamidofluorenones was synthesized, the study showing that some compounds have good antiproliferative activity, acting as type I topoisomerase inhibitors. It was found that the introduction of the linear alkyl group in the side chains resulted in better antiproliferative activity, comparing with compounds having a branched alkyl group or a bulky group. Compared to tertiary amino groups, the presence of secondary amino groups in the side chains is increasing the antiproliferative activity. The results indicated that the fluorenone fragment could be a potential pharmacophore for the design of antitumor compounds in the class of topoisomerase inhibitors IB [9].

Fluorenone derivatives have been also reported to possess potent antioxidant activity [10,11].

The choice of the carbamoyloximinic moiety in the structure of the compounds covered by this paper is also based on the fact that this moiety is found in the structure of other compounds with antibacterial and antifungal action, such as *N*-R-carbamoyl-2-adamantanoxime [12] or antimycobacterial, *N*,*N*-dimethylcarbamoyloxime 2-bromo-6-R-indeno[2,1-c]quinolin-7-one [13] or is also found in the structure of compounds known as acaricides, insecticides and/or nematocides (alanicarb, aldicarb, aldoxicarb, tirpate, oxamyl), as well as pesticides, such as *N*-methyl-*N*-[(*tert*-butylphenyl)sulfenyl] carbamoyloxime derivatives [14].

This structural feature of carbamate oxime, present in the molecule of some compounds, contributes to the improvement of their pharmacological and pharmacokinetic properties [15].

This article is focused on O-aryl-carbamoyl-oxymino-fluoren derivatives combining several pharmacophoric fragments, i.e., the fluorenic tricyclic system, carbamoyl and oximinic groups. The new derivatives were obtained by refluxing 9*H*-fluoren-9-one oxime with arylisocyanates and were characterized by their physical constants and spectroscopic data.

The nanotechnological approaches are more and more present in most fields of human activity and offer great expectations for the infection control too, by providing solutions for novel antimicrobial strategies, effective against both planktonic and adherent microbes, or by the improvement of current ones [16]. Among nanostructures, metal oxide nanomaterials (e.g., zinc oxide, copper oxide, magnetite) have gain particular attention, due to their intrinsic antimicrobial activity [17,18], but also to their ability to be used as nanoshuttles for the delivery of antimicrobial compounds. Starting from this evidence, our aim was to investigate the potential of magnetite@citrate core@shell nanostructures to improve the antimicrobial properties of newly synthesized fluorenones. These nanoparticles have many advantages for biomedical applications, such as increased biocompatibility and capacity to incorporate and release many therapeutic compounds, thus decreasing the required active concentration and the potential toxic effects [19,20,21,22]. These magnetic nanostructures should be exploited because the low dimensionality of these carriers can assure higher internalisation into the cells [23].

The antimicrobial activity was evaluated against bacterial and fungal strains, in planktonic and biofilms growth state, using quantitative assays, to determine the minimum inhibitory concentration (MIC) and the minimum biofilm eradication concentration (MBEC).

## 2. Results

### 2.1. Chemistry and Spectral Data

**Scheme 1:**
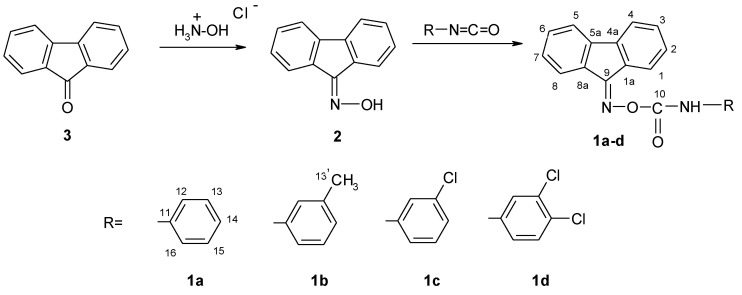
Synthesis of the new derivatives (**1a**–**d**).

The synthesis of O-aryl-carbamoyl-oxymino-fluorene derivatives (**1a**–**d**) involved the following steps: (i) preparing 9*H*-fluoren-9-one oxime (**2**), by refluxing 9-fluorenone (**3**) with hydroxylamine hydrochloride, in a molar ratio of 1: 1.3, the reaction medium being methanol; (ii) preparation of the new O-aryl-carbamoyl-oxymino-fluoren derivatives by refluxing 9*H*-fluoren-9-one oxime with arylisocyanates, in a molar ratio of 1: 1, in anhydrous tetrahydrofuran (Scheme 1).

Optimal reaction conditions were established to obtain the new compounds with high purity and good yields.

In NMR spectra, chemical shifts were recorded as δ, in parts per million (ppm), relative to tetramethylsilane as internal standard, and coupling constants (*J*) in Hertz. The following standard abbreviations indicating the multiplicity of signals were used: s (singlet), d (doublet), t (triplet), m (multiplet), dd (double doublet), td (triple doublet), and br (broad signal). ^1^H-NMR data are reported in the following order: chemical displacements, multiplicity, coupling constants, number of protons and signal/atom assignment. The order of the ^13^C-NMR data was chemical shifts and signal/atom assignment.

The ^1^H-NMR spectra of the new compounds (**1a**–**d** Figures S3, S5, S7 and S9) showed a broad singlet signal at 8.5–8.60 ppm attributed to the -NH-from the side chain; the doublet at 8.46–8.60 ppm was assigned to the H-1 proton, and this is the most deshielded signal of the fluorene nucleus protons. The triplet signal corresponding to the H-3 or H-2 proton of the fluorene nucleus appears in NMR spectra at δ = 7.30–7.37 ppm, being the most shielded signal.

The ^13^C-NMR spectra of the new compounds (Figures S4, S6, S8 and S10) showed signals at 155.92–156.53 ppm due to the C-9 carbon. This is the most deshielded signal of carbon atoms in fluorene fragment, and is followed by the signal of C-4a, which was registered in the range 141.51–142.48 ppm. The most shielded signal is that of C-5 or C-8 from 120.16 to 120.32 ppm. The signal at 151.16–151.75 ppm due to C-10 carbon is the most deshielded signal of a carbon atom in the side chain. All the signals were in complete agreement with the analysed structures.

The IR spectra were obtained using the ATR technique and were given as: w—weak band; m—medium band; s—intense band; vs—very intense band. The obtained results confirm both the structure of the new compounds and the intermediates used, as well as the performed synthesis.

### 2.2. Characterization of the Obtained Nanostructures

#### 2.2.1. Transmission Electron Microscopy (TEM)

**Figure 3:**
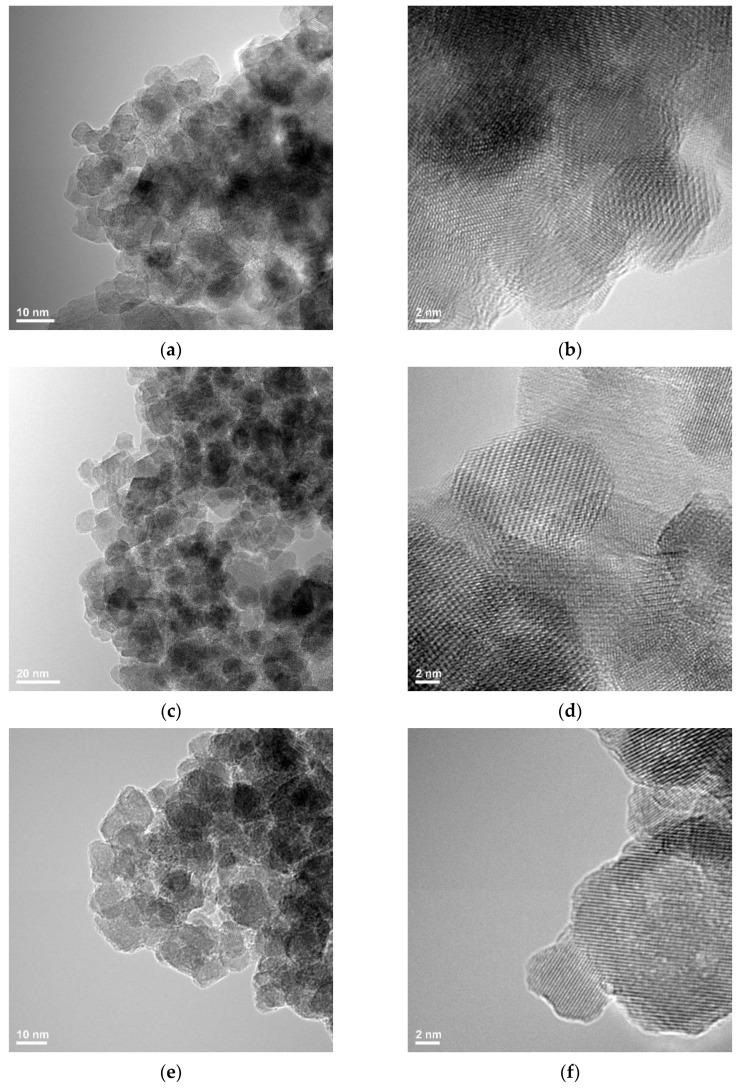

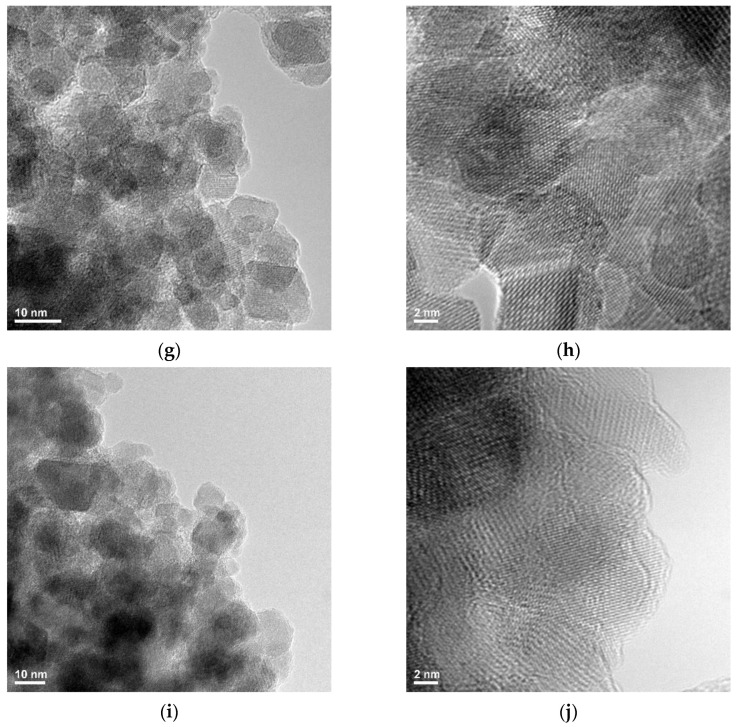
Transmission Electron Microscopy images for magnetic samples loaded with the fluorene derivatives: (**a**,**b**) Fe_3_O_4_@citrate, (**c**,**d**) Fe_3_O_4_@ citrate@1a, (**e**,**f**) Fe_3_O_4_@ citrate@1b, (**g**,**h**) Fe_3_O_4_@ citrate@1c, (**i**,**j**) Fe_3_O_4_@ citrate@1d.

In the case of the control sample (magnetite nanoparticles stabilized with citrate), agglomerations of nanoparticles with polyhedral shapes with dimensions in the range of 5–15 nm are observed. Because all the samples loaded with biologically active agents are obtained starting from these nanoparticles, the size and shape are not changing considerably, but the agglomeration tendency can be slightly different, especially compared with the pure magnetic nanoparticles stabilized with citrate. In all the cases, polyhedral nanoparticles are visible with particles of 5–10 nm except the samples **Fe_3_O_4_@ citrate@1a** which seem to be of 10–15 nm. Among all these samples, the sample **Fe_3_O_4_@citrate@1d** seems to be the most agglomerated one (Figure 3).

#### 2.2.2. Vibrating Sample Magnetometer (VSM)

**Figure 4:**
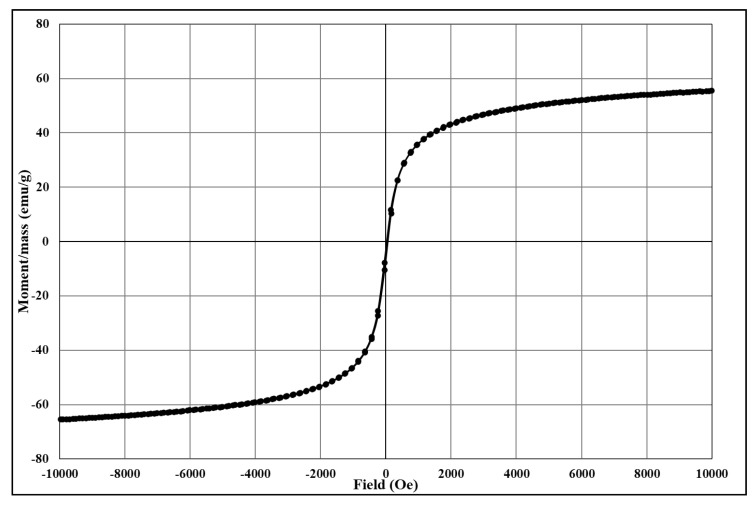
The magnetization/mass ratio for Fe_3_O_4_@citrate (reference sample).

**Figure 5:**
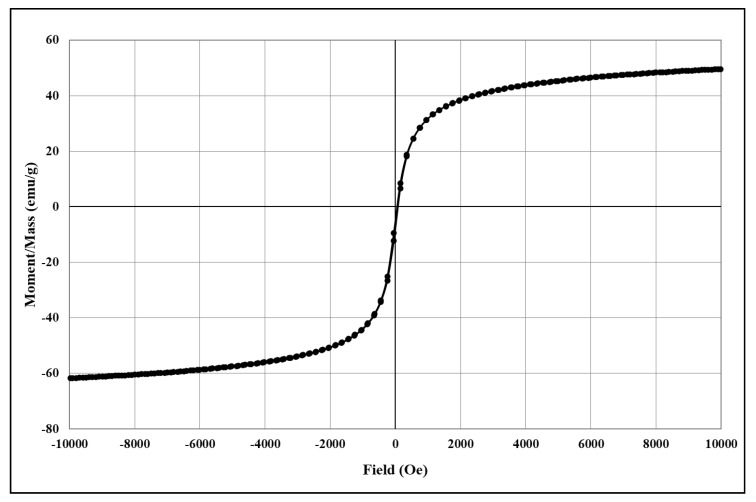
The magnetization/mass ratio for Fe_3_O_4_@citrate@1a.

**Figure 6:**
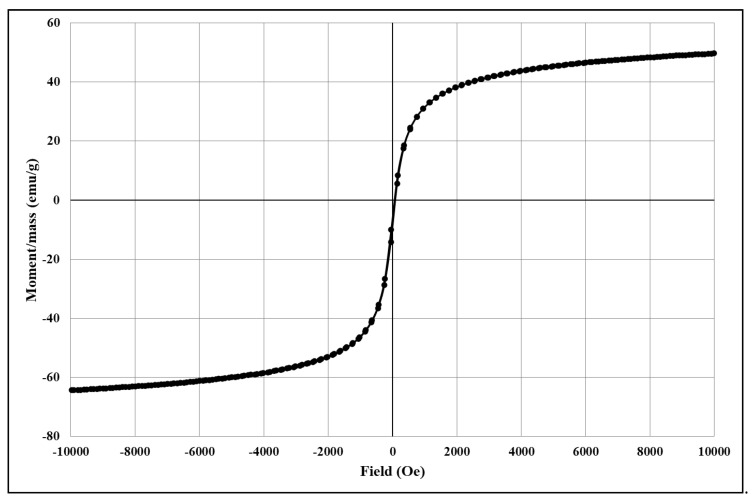
The magnetization/mass ratio for Fe_3_O_4_@citrate@1b.

**Figure 7:**
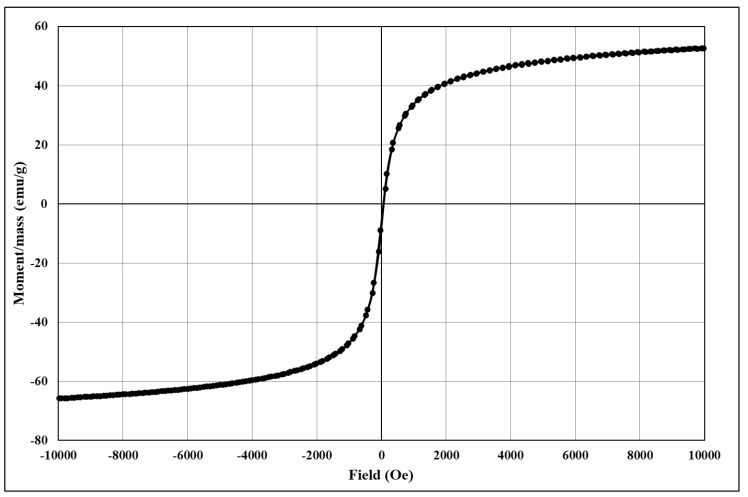
The magnetization/mass ratio for Fe_3_O_4_@citrate@1c.

**Figure 8:**
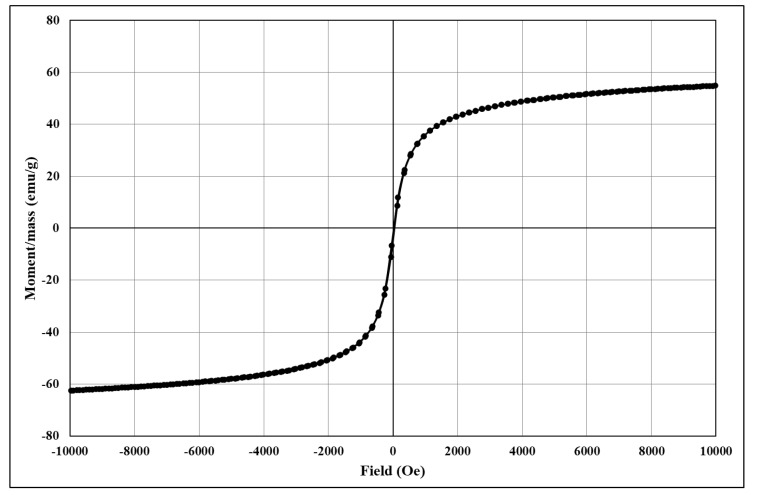
The magnetization/mass ratio for Fe_3_O_4_@citrate@1d.

The analysis of the degree of magnetization shows characteristic magnetization curves corresponding to magnetite and its derived core@shell homologues. The magnetization/mass ratio for magnetic core@shell structures loaded with the newly synthesised fluorene derivatives is presented below (Figure 4, Figure 5, Figure 6, Figure 7 and Figure 8).

The magnetisation/mass ratio of magnetite stabilized with citrate is 60.506 emu/g while the coercivity is 12.755Oe, and that of magnetite functionalized with bioactive compounds has slightly decreased being 55.650 (Fe_3_O_4_@citrate@1a), 56.986 (Fe_3_O_4_@citrate@1b), 59.233 (Fe_3_O_4_@citrate@1c) and 58.628 emu/g (Fe_3_O_4_@citrate@1d). The magnetisation decrease is due to the shielding capacity of the biologically active compounds **1a**–**d**.

#### 2.2.3. Fourier-Transform Infrared (FTIR) Spectroscopy

**Figure 9:**
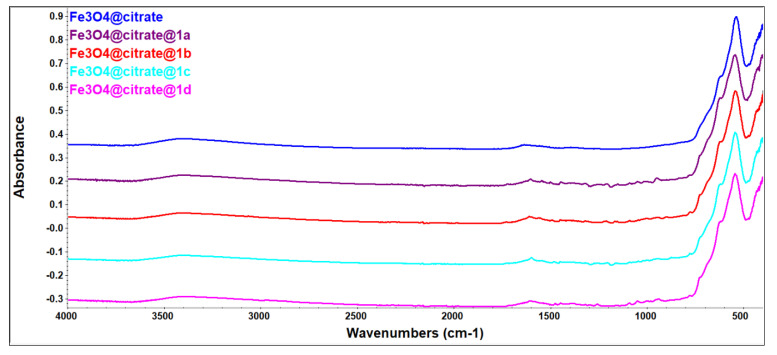
The FTIR spectra of magnetic samples loaded with fluorene derivatives.

The FTIR spectroscopy was used to identify the presence of functional groups of the Fe_3_O_4_ and the modifications by the presence of the newly synthesised fluorene derivatives loaded in magnetic core@shell nanostructures. Due to the loading of magnetite with the biological compounds **1a**–**d**, the FTIR spectra of these systems (Figure 9) highlight their absorption bands overlapped over the Fe_3_O_4_@citrate spectrum. Because the final systems contain 5% of the biological compounds **1a**–**d**, their contribution is marginal, explaining the very low intensity of the respective bands. The most important peak is assigned to the Fe-O vibration and is centred at 544 cm^−1^ which also prove the nanometric size of these nanoparticles [24]. It is worth mentioning that these peaks are shifted, which means that chemical interactions occur in the preparation step. Because the citrate content is low, its characteristic peaks have low intensity and can be only visualised as a shoulder or after deconvolution.

### 2.3. Antimicrobial and Antibiofilm Activity of the Obtained Hybrid Nanostructures Based on Magnetite Functionalized with the Compounds ***1a**–**d***

The antimicrobial activity of the newly synthesized compounds was evaluated against three microbial strains, respectively: *Escherichia coli* ATCC 25922, *Staphylococcus aureus* ATCC 1026, and *Candida albicans* ATCC 10231, in suspension and adhered as biofilms developed on an inert substrate.

#### 2.3.1. Antimicrobial Activity against Planktonic Microbial Cells

The tested compounds embedded in the core@shell magnetite nanoparticles showed an improved antibacterial and antifungal activity in the majority of cases, as revealed by the MIC values, ranging from 0.156 to 4 mg/mL in case of bare compounds and between 0.0625 and 2 mg/mL for the embedded ones, i.e., 2–8 times lower (Table 1). All nanoparticulate complexes proved the same efficiency against the *Candida albicans* strain, while the most active one was Fe_3_O_4_@citrate@1d against *Staphylococcus aureus* strain, exhibiting the lowest MIC value (MIC 0.0625 mg/mL).

#### 2.3.2. Anti-Adherence Activity of the Tested Compounds

All tested free compounds inhibited the development of the bacterial and fungal biofilms, the MBEC values being between 0.0625 and 1 mg/mL, up to twice times lower than the corresponding MIC values, except the *E. coli* strain (Table 2). It is worth noting the increased susceptibility of *Staphylococcus aureus* biofilms to compound **1d**, of *Escherichia coli* biofilm to **1c** and *Candida albicans* to **1a**–**c**, but especially to **1b**.

In exchange, the nanoparticulate systems have generally exhibited a decreased anti-biofilm efficiency, excepting compounds **1a**, **1b,** and **1c** against *Staphylococcus aureus* and **1c** against *Escherichia coli.*

## 3. Discussion

The main purpose of this study was to synthesize and structurally and biologically characterize novel derivatives with antimicrobial potential, as well as to increase their bioactivity by using a nanotechnological approach. Fluorene, a tricyclic aromatic hydrocarbon, is found in many drugs and many fluorene derivatives have been shown to exhibit antimicrobial activities against Gram-positive and Gram-negative bacterial strains, including mycobacteria, as well as on yeasts and molds [6,7,8,12]. One of the main challenges in the development of novel antimicrobials is related to their efficiency against biofilm embedded microorganisms, which are responsible of an important fraction of the total number of infections, with a clear tendency for chronicization and persistence, raising difficult therapeutic issues and requiring high doses or complex regimens, combining multiple antimicrobial agents to surpass the high phenotypic resistance of bacterial biofilms. The nanotechnological approaches seem to offer a promising potential for fighting infections produced by resistant or biofilm forming microorganisms, due to their antimicrobial activity, mediated by multiple mechanisms or to their ability to improve the activity of current antimicrobials [16,17,18]. Nowadays, important progress has been recorded regarding the use of magnetic nanoparticles for biomedical applications. A special focus is given to magnetic nanoshuttles that are intensively studied both for their intrinsic antimicrobial features, as well for developing new targeting and controlled delivery systems for natural and synthetic antimicrobial compounds. Due to the magnetic behaviour, these nanoparticles can act as potent carriers for targeted drug, including intracellular, delivery, bioaccumulation being controlled by the externally applied magnetic field. According to the literature [25], it is expected that these nanoparticles are also suitable for MRI imaging, so multifunctional systems can be designed with both therapeutic and diagnostic capabilities, also having the advantage of a very good biocompatibility [19,20,21,22].

Taking into account the promising potential of fluorine derivatives as broad antimicrobial agents, we have used in our study the 9*H*-fluoren-9-one oxime as key intermediate, which was treated with phenyl isocyanate derivatives in anhydrous tetrahydrofuran to obtain the novel compounds. The yields were good and the reaction times were acceptable. The obtained compound structure was elucidated by the NMR and IR spectra which confirmed both the structure of the new derivatives and of the intermediates.

Then, we have investigated the potential of magnetite@citrate core@shell nanostructures to improve the antimicrobial properties of newly synthesized fluorenones.

The nanostructured systems containing Fe_3_O_4_@ citrate and the obtained fluorine derivatives were characterized by TEM, revealing polyhedral shapes and a size of 5–15 nm. According to the literature, these nanoparticles have similar size as those produced by Favela-Camacho et al. [26] using two methods, namely the fast injection co-precipitation and the reflux coprecipitation method.

Being known that magnetisation of the magnetite nanoparticles is a size-dependent property, we have further analyzed the degree of magnetization as well as the magnetization/mass ratio and comparing our data with those published by others, in the same Fe_3_O_4_@citrate system, we were able to evaluate the size of these nanoparticles, at bulk level. According to the work published by Shen et al. [25] our Fe_3_O_4_@citrate nanoparticles having a normalised magnetisation of 60.506 emu/g fit well between the nanoparticles of 4.2 and 13.8 nm (their magnetisation being 32.00 and 82.6 emu/g), thus proving that the TEM images are relevant for the bulk magnetic powder.

Further, the loading of magnetite with the obtained derivatives **1a**–**d** was confirmed by the FTIR spectra.

The antimicrobial activity was evaluated against Gram-negative (*Escherichia coli*), Gram-positive (*Staphylococcus aureus*) and fungal (*Candida albicans*) strains, grown in planktonic and biofilms state, using quantitative assays allowing us to establish the MIC and MBEC values for the bare compounds, comparatively with the compounds included in core@shell magnetite nanoshuttles. The used strains are traceable to ATCC (American Type Culture Collection) and are well characterized in terms of their susceptibility to a large range of antimicrobial agents.

Regarding the antimicrobial activity of the bare compounds, our results revealed that the nature of substituents on the aryl moiety are influencing the antimicrobial spectrum and intensity of the inhibitory effect on the microbial growth. The electron-withdrawing inductive effect, known as the -I effect, of chlorine atoms enhances the activity of compound **1d** against *S. aureus* ATCC 1026 in planktonic and biofilm growth state (MIC value 0.156 mg/mL; MECB value 0.019 mg/mL). All tested compounds inhibited the development of bacterial and fungal biofilms, with very low MBEC values, compared to the corresponding MIC ones.

Further, our results have shown that the incorporation of the synthesized compounds into magnetite nanoparticles led to an improved antibacterial and antifungal activity of the tested bioactive substances the MIC values being 2–8 times lower. The most statistically significant results were recorded for compound **1c** (*p* < 0.0001, unpaired *t*–test).

However, the incorporation of the synthesized compounds into the nanoparticulate systems has generally decreased their anti-biofilm efficiency. In general, the cells associated in biofilms are more difficult to be destabilized. The different results can be explained considering different mechanisms of internalization (of the active agents) in the planktonic versus biofilm associated cells. In biofilms, the matrix and external cell layers are protecting the internal cells and the nanoparticles loaded with the active agents collide with these external biofilm layers. In the case of planktonic cells, the internalization process can be achieved by both diffusion of the free agent, but also as a consequence of the collision between the nanoparticulated shuttle and the free cells.

## 4. Materials and Methods

### 4.1. General Information

All chemicals and solvents were purchased from Merck (Darmstadt, Germany), Fluka (Buchs, Switzerland), Sigma–Aldrich (St. Louis, MO, USA), Roth (Karlsruhe, Germany) and Alfa Aesar (Kandel, Germany) and were of commercial quality and they were used as received. THF was dried by refluxing 1 h over potassium hydroxide and then distillated at normal pressure.

The synthesis of Fe_3_O_4_@citrate magnetic nanoparticles was performed using sodium hydroxide (Sigma-Aldrich), double iron and ammonium sulphate (Roth), iron chloride (Sigma-Aldrich), anhydrous trisodium citrate (Alfa Aesar), silver nitrate (Sigma-Aldrich), and ethyl alcohol (Sigma-Aldrich).

The ^1^H-NMR and the ^13^C-NMR spectra of the O-aryl-carbamoyl-oxymino-fluorene derivatives are found in the supplementary material. 

### 4.2. Chemistry

Melting points were determined on an Electrothermal 9100 (Bibby Scientific Ltd., Stone, UK), in open capillaries.

The structures of the original and intermediate compounds were determined by IR, ^1^H-NMR and ^13^C-NMR spectral analysis. Chemical shifts for hydrogen and carbon atoms have also been confirmed by 2D-NMR experiments.

IR spectra were recorded on a Bruker Vertex 70 FT-IR spectrometer (Bruker Corporation, Billerica, MA, USA).

^1^H-NMR and ^13^C-NMR spectra were recorded in deuterated chloroform (CDCl_3_) or DMSO-d6, on a Bruker Fourier 300 MHz instrument (Bruker Corporation, Billerica, MA, USA) operating at 300.0 MHz for ^1^H-NMR and at 75.0 MHz for ^13^C-NMR and on a Bruker Avance III 500 MHz instrument (Bruker Corporation, Billerica, MA, USA) operating at 500 MHz for proton and 125 MHz for carbon).

#### 4.2.1. Synthesis Procedure of the 9H-Fluoren-9-one Oxime and Spectral Data

In a round-bottomed, 4-necked flask fitted with a shaker, thermometer, water-cooled condenser, and drip funnel, are placed 5 g of 9-fluorenone (molecular weight (Mw) 180.19) (0.028 mol) dissolved in 20 mL of methanol, over which gradually add, under stirring, 5.5 g of sodium hydroxide (Mw 39.99). Over the mixture, gradually add, with stirring, at 75–80 °C, 2.5 g of hydroxylamine hydrochloride (Mw 69.49) (0.036 mol), dissolved in 10 mL of methanol. Then, the reaction mixture was refluxed for 5 h. After cooling to room temperature, a mixture of 12.5 mL hydrochloric acid and 27 mL of water is gradually added until pH 8, when the oxime precipitates. The mixture stirs at room temperature for one hour, and then, the oxime is filtered under low pressure and washed thoroughly on the filter with water. After isolating the precipitate, it was dried, then purified from xylene.

Result 4.80 g of compound (Mw 195.22), crystallized, yellow, in 88.5% yield, T.t. 194.3–195.2 °C, soluble in cold ethyl acetate, DMF, DMSO, pyridine and in hot methanol, ethanol, isopropanol, isobutanol, chloroform and xylene, insoluble in hexane and water.

^1^H-NMR (DMSO-d6, δ ppm, *J* Hz): 12.58 (s, N-OH); 8.37 (d, *J* = 7.3 Hz, 1H, H-1); 7.90 (d, *J* = 7.6Hz, 1H, H-4); 7.85 (d, *J* = 7.6, Hz, 1H, H-5 or H-8); 7.73 (d, *J* = 7.3 Hz, 1H, H-5 or H-8); 7.32–7.54 (m, 4H, H-2, H-3, H-6, H-7).

^13^C-NMR (DMSO-d6, δ ppm): 150.96 (C-9); 140.13 (C-4a); 139.25 (C-5a); 135.28 (C-8a); 130.73 (C-1a); 129.65 (C-6 or C-7); 129.60 (C-6 or C-7); 128.48 (C-1); 128.35 (C-2); 127.98 (C-3); 120.80 (C-4); 120.38 (C-5 or C-8); 120.31 (C-5 or C-8).

FT-IR (solid in ATR, ν cm^−1^): 3166w; 3039w; 2617m; 1642m; 1603m; 1588vs; 1485s; 1433m; 1319m; 1167s; 1090m; 1001s; 947s; 873s; 737w; 676vs; 621vs.

#### 4.2.2. Synthesis Procedure of the 9-(Phenylcarbamoyloxymino)fluorene (**1a**) and Spectral Data

Briefly, 0.59 g of 9*H*-Fluoren-9-one oxime (Mw 195.22) (0.003 mol) solubilized in 10 mL of anhydrous tetrahydrofuran is introduced in a round-bottomed flask. A solution of 0.36 g of phenyl isocyanate (Mw 119.12) (0.003 mol) in 10 mL of anhydrous tetrahydrofuran is added. The reaction mixture was refluxed on a water bath for 52 h, then chilled, and the solvent was removed under reduced pressure. The obtained product is purified from ethyl acetate.

The result is 0.60 g of crystallized yellow compound (Mw 314.33), with a yield of 63.2% toward oxime, T.t. 156.9–159^0^C, soluble in cold pyridine, DMF, DMSO, chloroform, soluble in hot methanol, ethanol, isopropanol, isobutanol, ethyl acetate, xylene, insoluble in hexane and water.

^1^H-NMR (CDCl_3_, δ ppm, *J* Hz): 8.56 (br s, 1H, NH); 8.46 (d, *J* = 7.5 Hz, 1H, H-1); 7.79 (d, *J* = 7.5 Hz, 1H, H-4); 7.61–7.56 (m, 4H, H-5, H-8, H-12, H-16); 7.45 (td, *J* = 7,5 Hz, *J* = 1.0 Hz, 1H, H-6 or H-7); 7.44 (td, *J* = 7.5 Hz, *J* = 1.1 Hz, 1H, H-7 or H-6); 7.41 (t, *J* = 7.8 Hz, 2H, H-13, H-15); 7.32 (t, *J* = 7.5 Hz, 1H, H-2); 7.30 (t, *J* = 7.5 Hz, 1H, H-3); 7.16 (t, *J* = 7.8 Hz, 1H, H-14).

^13^C-NMR (CDCl_3_, δ ppm): 155.97 (C-9); 151.75 (C-10); 142.31 (C-4a); 141.47 (C-5a); 136.80 (C-8a); 133.93 (C-1a); 132.84 (C-6 or C-7); 131.82 (C-6 or C-7); 130.99 (C-1); 129.72 (C-11); 129.17 (C-13, C-15); 128.83 (C-2); 128.23 (C-3); 124.48 (C-14); 122.55 (C-4); 120.33 (C-5 or C-8); 120.16 (C-5 or C-8); 119.79 (C-12, C-16).

FT-IR (solid in ATR, ν cm^−1^): 3267m; 3138w; 3064w; 2962w; 1724vs; 1603s; 1543vs; 1501m; 1446s; 1315m; 1208vs; 1017m; 952vs; 837w; 809w; 788w; 749m; 729m; 688m; 646w.

#### 4.2.3. Synthesis Procedure of the 9-((3-Methyl-phenyl)carbamoyloxymino)fluorene (**1b**) and Spectral Data

The compound was prepared by the above method, requiring 0.399 g of 3-methylphenyl isocyanate (Mw133.15, d_4_^20^ = 1.033; 0.003 mol).

0.56 g of compound (Mw 328.36) were obtained after recrystallization from ethyl acetate, with a yield of 56.4% toward oxime, T.t. 171.2–174.3 °C, soluble in cold pyridine, DMF, DMSO, chloroform, soluble in hot methanol, ethanol, isopropanol, isobutanol, ethyl acetate, xylene, insoluble in hexane and water.

^1^H-NMR (CDCl_3_, δ ppm, *J* Hz): 8.51 (br s, 1H, NH); 8.48 (d, *J* = 7.5 Hz, 1H, H-1); 7.81 (d, *J* = 7.5 Hz, 1H, H-4); 7.60 (d, *J* = 7.5 Hz, 1H, H-5 or H-8); 7.59 (d, *J* = 7.5 Hz, 1H, H-5 or H-8); 7.48 (t, *J* = 7.5 Hz, 1H, H-6 or H-7); 7.45 (t, *J* = 7.5 Hz, 1H, H-6 sau H-7); 7.41 (br s, 1H, H-12); 7.40 (d, 1H, H-16); 7.34 (t, *J* = 7.5 Hz, 1H, H-2 or H-3); 7.31 (t, *J* = 7.5 Hz, 1H, H-2 or H-3); 7.28 (t, *J* = 8.2 Hz, 1H, H-15); 6.99 (d, *J* = 8.2 Hz, 1H, H-14); 2.40 (s, 3H, H-13′).

^13^C-NMR (CDCl_3_, δ ppm): 155.92 (C-9); 151.72 (C-10); 142.34 (C-4a); 141.51 (C-5a); 139.14 (C-13); 136.71 (C-8a); 134.00 (C-1a); 132.85 (C-6 or C-7); 131.92 (C-11); 131.80 (C-6 or C-7); 131.08 (C-1); 129.00 (C-2); 128.86 (C-15); 128.25 (C-3); 125.32 (C-14); 122.58 (C-4); 120.43 (C-16); 120.34 (C-5 or C-8); 120.17 (C-5 or C-8); 116.91 (C-12); 21.50 (C-13′).

FT-IR (ATR in solid, ν cm^−1^): 3259m; 3149w; 3084w; 3054w; 3022w; 2920w; 1729vs; 1613s; 1555s; 1491m; 1449m; 1316m; 1211vs; 1169m; 1155m; 1028m; 968s; 900s; 780m; 728m; 690m.

#### 4.2.4. Synthesis Procedure of the 9-((3-Chloro-phenyl)carbamoyloxymino)fluorene (**1c**) and Spectral Data

The synthesis follows the procedure described for the preparation of the compound **1a**, using 0.46 g of 3-chlorophenyl isocyanate (Mw = 153.57, d_4_^20^ = 1.269; 0.003 mol).

The result was 0.7 g of compound (Mw 348.77), after recrystallization from ethyl acetate, in 66.4% yield toward oxime, T.t. 155.7–158.3 °C, soluble in cold pyridine, DMF, DMSO, chloroform, soluble in hot methanol, ethanol, isopropanol, isobutanol, ethyl acetate, xylene, insoluble in hexane and water.

^1^H-NMR (CDCl_3_, δ ppm, *J* Hz): 8.59 (br s, 1H, NH, deuterable); 8.60 (d, *J* = 7.5 Hz, 1H, H-1); 7.82 (d, *J* = 7.5 Hz, 1H, H-4); 7.66 (t, *J* = 1.9 Hz, 1H, H-12); 7.59 (d, *J* = 7.5 Hz, 1H, H-5 or H-8); 7.58 (d, *J* = 7.5 Hz, 1H, H-5 or H-8); 7.56 (t, *J* = 7.5 Hz, 1H, H-6 or H-7); 7.54 (m, 1H, H-14); 7.50 (t, *J* = 7.5 Hz, 1H, H-6 or H-7); 7.40 (t, *J* = 7.5 Hz, 1H, H-2 or H-3); 7.37 (t, *J* = 7.5 Hz, 1H, H-2 or H-3); 7.36 (t, *J* = 8.1 Hz, 1H, H-15); 7.19 (dd, *J* = 8.1 Hz, *J* = 1.9 Hz, 1H, H-16).

^13^C-NMR (CDCl_3_, δ ppm): 156.31 (C-9); 151.16 (C-10); 142.45 (C-4a); 141.60 (C-5a); 138.02 (C-8a); 134.88 (C-13); 133.83 (C-1a); 133.08 (C-6 or C-7); 132.04 (C-6 or C-7); 131.13 (C-1); 130.30 (C-15); 129.73 (C-11); 128.96 (C-2); 128.34 (C-3); 124.57 (C-16); 122.64 (C-4); 120.45 (C-5 or C-8); 120.28 (C-5 or C-8); 119.80 (C-12); 117.74 (C-14).

FT-IR (ATR in solid, ν cm^−1^): 3251w; 3189w; 3125w; 3081w; 2962w; 1728vs; 1596vs; 1545s; 1481m; 1450m; 1415w; 1306m; 1204vs; 1022m; 968vs; 874s; 812w; 779m; 726m; 682m.

#### 4.2.5. Synthesis Procedure of the 9-((3,4-Dichloro-phenyl)carbamoyloxymino) fluorene (**1d**) and Spectral Data

The same synthetic procedure was followed as for the compound **1a**, using 0.564 g of 3,4-dichlorophenyl isocyanate (Mw 188.01; 0.003 mol).

0.75 g of compound (Mw 383.23) is obtained after purification from ethyl acetate, with a yield of 64.8% toward oxime, T.t. 157.9–161.2 °C, soluble in cold pyridine, DMF, DMSO, chloroform, soluble in hot methanol, ethanol, isopropanol, isobutanol, ethyl acetate, xylene, insoluble in hexane and water.

^1^H-NMR (CDCl_3_, δ ppm, *J* Hz): 8.60 (br s, 1H, NH, deuterable); 8.53 (d, *J* = 7.7 Hz, 1H, H-1); 7.80 (d, *J* = 7.7 Hz, 1H, H-4); 7.78 (d, *J* = 2.3 Hz, 1H, H-12); 7.62 (d, *J* = 7.2 Hz, 1H, H-5 or H-8); 7.61 (d, *J* = 7.2 Hz, 1H, H-5 or H-8); 7.43–7.51 (m, 4H, H-6, H-7, H-15, H-16); 7.35 (t, *J* = 7.7 Hz, 1H, H-2); 7.32 (t, *J* = 7.7 Hz, 1H, H-3).

^13^C-NMR (CDCl_3_, δ ppm): 156.53 (C-9); 151.45 (C-10); 142.48 (C-4a); 141.63 (C-5a); 136.37 (C-8a); 133.74 (C-1a); 133.05 (C-14); 133.16 (C-6 or C-7); 132.13 (C-6 or C-7); 131.11 (C-1); 130.73 (C-16); 129.69 (C-11); 128.97 (C-2); 128.35 (C-3); 127.86 (C-13); 122.66 (C-4); 121.41 (C-12); 120.48 (C-5 or C-8); 128.32 (C-5 or C-8); 118.37 (C-15).

FT-IR (ATR in solid, ν cm^−1^): 3366m; 1768m; 1597w; 1574w; 1504vs; 1477m; 1450w; 1380w; 1320w; 1287w; 1235w; 1185w; 1025m; 956m; 920w; 848w; 781w; 724m; 685w.

### 4.3. Synthesis of Fe_3_O_4_ Nanoparticles and of Magnetic Nanoparticles Loaded with Biologically Active Compounds

Magnetic nanoparticles were synthesized by the coprecipitation method [27,28], using double iron and ammonium sulphate hexahydrate and iron chloride (III) as precursors and anhydrous trisodium citrate as a stabilizing agent. After the Fe_3_O_4_@citrate magnetic nanoparticles were synthesized, they were loaded with bioactive compounds (**1a**–**d**) by grinding 100 mg of Fe_3_O_4_@citrate nanopowder with a solution formed by dissolving 5 mg of active substance in 1 mL of ethanol. To obtain a homogeneous system, this suspension was milled until the total evaporation of the solvent. For complete drying, these systems were finally dried in vacuum (2mbar) for ~1 h, leading to the desired nanopowders denoted with Fe_3_O_4_@citrate@1a–d. It is worth to mention that this methodology of loading of the biologically active agents is important because can be used even for the loading of hydrophobic biologically active agents the loading efficiency is maximal.

The obtained nanocomposites have been characterized by TEM, VSM, and FTIR.

Transmission electron microscopy (TEM) images corresponding to Fe_3_O_4_@citrate magnetic nanoparticles were obtained using a high-resolution Tecnai G2 F30 S-TWIN transmission electron microscope equipped with a Selected Area Electron Diffraction (SAED) module. The microscope operated in bright-field transmission mode at a voltage of 300 kV, with a punctual and line resolution of 2 Å and 1 Å respectively.

The Fourier-transform infrared spectroscopy (FT-IR) investigation of the magnetic carrier was done using the Nicolet iS50R spectrometer. Measurements were performed at room temperature using the total reflection attenuation module (ATR), with 32 sample scans between 4000 and 400 cm^−1^ at a resolution of 4 cm^−1^, with the scanning time being 47 s. The recording and the further processing and analysis of the data were possible by connecting the spectrometer to data acquisition and processing unit through the Omnic program.

The magnetic behaviour of the Fe_3_O_4_@citrate samples loaded with the fluorene derivatives was evaluated with the vibrating sample magnetometer (VSM) at 25 ± 2 °C using the 7400 Series VSM equipment manufactured by LakeShore.

### 4.4. Microbiological Assays

The antimicrobial activity of the newly synthesized compounds was evaluated against 5 microbial strains, respectively: *Escherichia coli* ATCC 25922, *Staphylococcus aureus* ATCC 1026, and *Candida albicans* ATCC 10231, in suspension and adhered as biofilms developed on an inert substrate. All strains tested are part from Microorganism Collection of Department of Microbiology, Faculty of Biology & Research Institute of the University of Bucharest.

#### 4.4.1. Minimum Inhibitory Concentration (MIC) Assay

The determination of MIC was performed by the method of serial binary microdilutions in Mueller Hinton broth, distributed in 96-well microplates. We tested ten concentrations of each compound (with values between 5 and 0.009 mg/mL) obtained after serial binary dilutions in a final volume of 100 μL of a medium, obtained from a stock solution of 10 mg/mL in dimethyl sulfoxide (DMSO). We have used the antibiotic tetracycline and the antifungal agent fluconazole as controls. Subsequently, the wells were seeded with 20 μL 0.5 MacFarland microbial suspension. The microbial culture cultivated in the absence of the test compound was used as growth control, and the negative one by the sterile culture medium. The microplates were incubated at 37 °C for 24 h, the MIC value is set at the level of the last well with clear, transparent content, with an appearance like the negative control. The assays were performed in triplicate, in order to confirm the MIC value [29].

#### 4.4.2. Anti-Biofilm Activity of the Tested Compounds (Minimal Biofilm Eradication Concentration -MBEC)

It was performed by the microtiter method, using the microplates used to determine the MIC values. For this purpose, the wells were washed two to three times with sterile saline, after which the biofilms adhered to the walls of the wells were fixed with 100 μL cold methanol for 5 min, stained with 1% alkaline solution of purple crystal, for 15 min, and then resuspended with 33% acetic acid. The MBEC was established as the last concentration of the compound at which was observed the decrease at 490 nm of the absorbance value, compared to the positive control. The assays were performed in triplicate in order to confirm the MBEC value [30].

## 5. Conclusions

A series of O-aryl-carbamoyl-oxymino-fluorene derivatives have been synthesized using the target compound 9*H*-fluoren-9-one oxime and were characterized by their physical constants. The structures of the synthesized compounds have been established based on their spectral data.

These novel synthesized derivatives containing 9*H*-fluorene scaffold exhibited inhibitory activity against planktonic and biofilm forms of selected bacteria. The bioactivity against planktonic cells has been significantly improved by the incorporation of the obtained compound in stabilized iron oxide nanoparticles. These systems were proven to have improved MIC values, but the MBEC values are higher comparing to the active compounds. Therefore, the obtained compounds and magnetic nanoparticles loaded with these compounds can be considered as antimicrobial agents with potential applications in the production of drugs and pharmaceuticals. It is worth continuing these studies in assessing magnetic triggering bioaccumulation and delivery.

## Figures and Tables

**Table 1:** Results of quantitative testing of antimicrobial activity against planktonic microbial strains and corresponding MIC values (mg/mL).

	Chemical Compound	1a/Fe_3_O_4_@citrate@1a	1b/Fe_3_O_4_@citrate@1b	1c/Fe_3_O_4_@citrate@1c	1d/Fe_3_O_4_@citrate@1d	Fe_3_O_4_@citrate	Fe_3_O_4_	DMSO	Positive Control
Microbial Strain									
***Staphylococcus aureus*** **ATCC 1026**		2/2	2/2	4/1 ***	**0.156/** **0.0625**	2	2	2	0.001
***Escherichia coli*** **ATCC 25922**		2/2	4/1	4/1	2/1	2	2	2	0.002
***Candida albicans*** **ATCC 10231**		2/0.5	4/0.5	4/0.5	4/0.5	1	1	1	0.004

*** *p* < 0.0001, Unpaired *t*–test (GraphPad Prism version 8.0.0 for Windows, GraphPad Software, San Diego, CA, USA, www.graphpad.com (accessed on 12 April 2021)).

**Table 2:** Antibiofilm activity test results and corresponding MBEC values (mg/mL).

	Chemical Compound	1a/Fe_3_O_4_@ citrate@1a	1b/Fe_3_O_4_@citrate@1b	1c/Fe_3_O_4_@citrate@1c	1d/Fe_3_O_4_@citrate@1d	Fe_3_O_4_@citrate	Fe_3_O_4_	DMSO	Positive Control
Microbial Strain									
***Staphylococcus aureus*** **ATCC 1026**		4/0.25	2/0.25	4/0.25	**0.0015/0.0625**	0.5	0.25	1	0.01
***Escherichia coli*** **ATCC 25922**		0.625/1	**0.0078**/0.5	1/0.25	0.0625/0.5	2	1	1	0.2
***Candida albicans*** **ATCC 10231**		0.312/0.25	**0.0078**/**0.0625**	0.0312/0.125	0.5	0.125	0.125	0.5	0.08

## Data Availability

Data available on request.

## Supplementary Materials

The ^1^H-NMR and the ^13^C-NMR spectra of the O-aryl-carbamoyl-oxymino-fluorene derivatives are found in the supplementary material (Figures S1–S10).

## Sample Availability

Samples of the compounds are not available from the authors.
