# Supplementary material for: New O-Aryl-Carbamoyl-Oxymino-Fluorene Derivatives with MI-Crobicidal and Antibiofilm Activity Enhanced by Combination with Iron Oxide Nanoparticles
Source: Molecules. 2021 May 18;26(10):3002. doi: 10.3390/molecules26103002 (PMC8158365; doi:10.3390/molecules26103002)
Supplement: Supplementary file 1 [file molecules-26-03002-s001.zip › molecules-1204615-supplementary.pdf]

molecules

Supplementary material

New O-Aryl-Carbamoyl-Oxymino-Fluorene Derivatives with MI-Crobicidal and Antibiofilm Activity Enhanced by Combination with Iron Oxide Nanoparticles

Ilinca Margareta Vlad [1], Diana Camelia Nuță [1], Robert Viorel Ancuceanu [2], Miron Teodor Caproiou [3], Florea Dumitrascu [3], Ioana Cristina Marinas [4], Mariana Carmen Chifiriuc [4,8], Luminita Gabriela Măruţescu [4,*], Irina Zarafu [5], Ioana Raluca Papacocea [6], Bogdan Ștefan Vasile [7], Adrian Ionuț Nicoară [7], Cornelia-Ioana Ilie [7], Anton Ficai [7,8,*] and Carmen Limban [1]

1 Department of Pharmaceutical Chemistry, Faculty of Pharmacy, "Carol Davila" University of Medicine and Pharmacy, 6 Traian Vuia, Bucharest, 020956, Romania; ilinca.vlad@drd.umfcd.ro (I.M.V.); diana.nuta@umfcd.ro (D.C.N.); carmen.limban@umfcd.ro (C.L.)
2 Department of Pharmaceutical Botany, Faculty of Pharmacy, "Carol Davila" University of Medicine and Pharmacy, 6 Traian Vuia, Bucharest, 020956, Romania; robert.ancuceanu@umfcd.ro
3 Costin D. Nenițescu" The Organic Chemistry Centre of Romanian Academy, 202B Splaiul Independenței, Bucharest, 060023, Romania; dorucaproiu@gmail.com (M.T.C.); fdumitra@yahoo.com (F.D.)
4 Department of Microbiology, Faculty of Biology & Research Institute of the University of Bucharest (ICUB), University of Bucharest, 060101 Bucharest, Romania; ioana.cristina.marinas@gmail.com (I.C.M.), carmen.chifiriuc@bio.unibuc.ro (M.C.C.)
5 Department of Organic Chemistry, Biochemistry and Catalysis, Faculty of Chemistry, University of Bucharest, 4-12 Regina Elisabeta, 030018 Bucharest, Romania; zarafuirina@yahoo.fr
6 Department of Physiology I, Faculty of Medicine, "Carol Davila" University of Medicine and Pharmacy, 8 Eroilor Sanitari, 050474, Bucharest, Romania; rpapacocea@gmail.com
7 Faculty of Applied Chemistry and Materials Science, University POLITEHNICA of Bucharest, 313 Spl. Independentei, 060042 Bucharest, Romania; bogdan.vasile@upb.ro (B.Ș.V.); adrian.nicoara@upb.ro (A.I.N.); cornelia_ioana.ilie@upb.ro (C.-I.I.)
8 Academy of Romanian Scientists, 3 Ilfov Street, 050045 Bucharest, Romania
* Correspondence: luminita.marutescu@bio.unibuc.ro (L.G.M.); anton.ficai@upb.ro (A.F.)

Abstract: Antimicrobial resistance is one of the major public health threats at global level, urging the search for new antimicrobial molecules. The fluorene nucleus is a component of different bioactive compounds, exhibiting diverse pharmacological actions. The present work describes the synthesis, chemical structure elucidation and bioactivity of new O-aryl-carbamoyl-oxymino-fluorene derivatives and the contribution of iron oxide nanoparticles to enhance the desired biological activity. The antimicrobial activity assessed against three bacterial and fungal strains, in suspension and biofilm growth state, using quantitative assay, revealed that the nature of substituents on the aryl moiety are determinant for both the spectrum and intensity of the inhibitory effect. The electron-withdrawing inductive effect of chlorine atoms enhanced the activity against planktonic and adhered *Staphylococcus aureus*, while the +I effect of methyl group enhanced the anti-fungal activity against *Candida albicans* strain. The magnetite nanoparticles have substantially improved the antimicrobial activity of the new compounds against planktonic microorganisms. The obtained compounds, as well as the magnetic core@shell nanostructures loaded with these compounds have a promising potential for the development of novel antimicrobial strategies.

Keywords: 9-fluorenone; 9*H*-fluoren-9-one oxime; O-aryl-carbamoyl-oxymino-fluorene derivatives; antimicrobial; antibiofilm; iron oxide nanoparticles

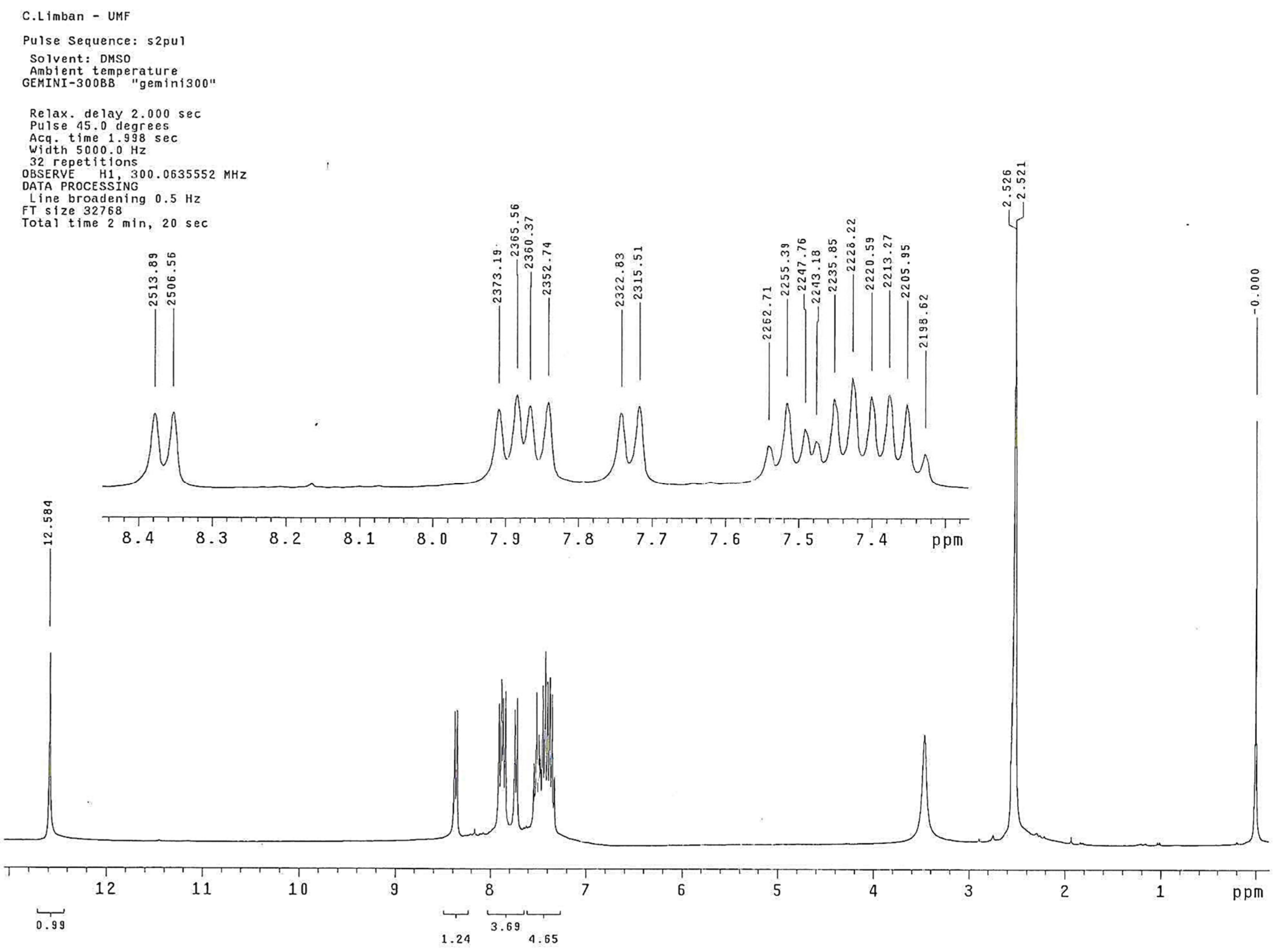

Figure S1. The ^{1}H- NMR spectra of the 9H-Fluoren-9-one Oxime.

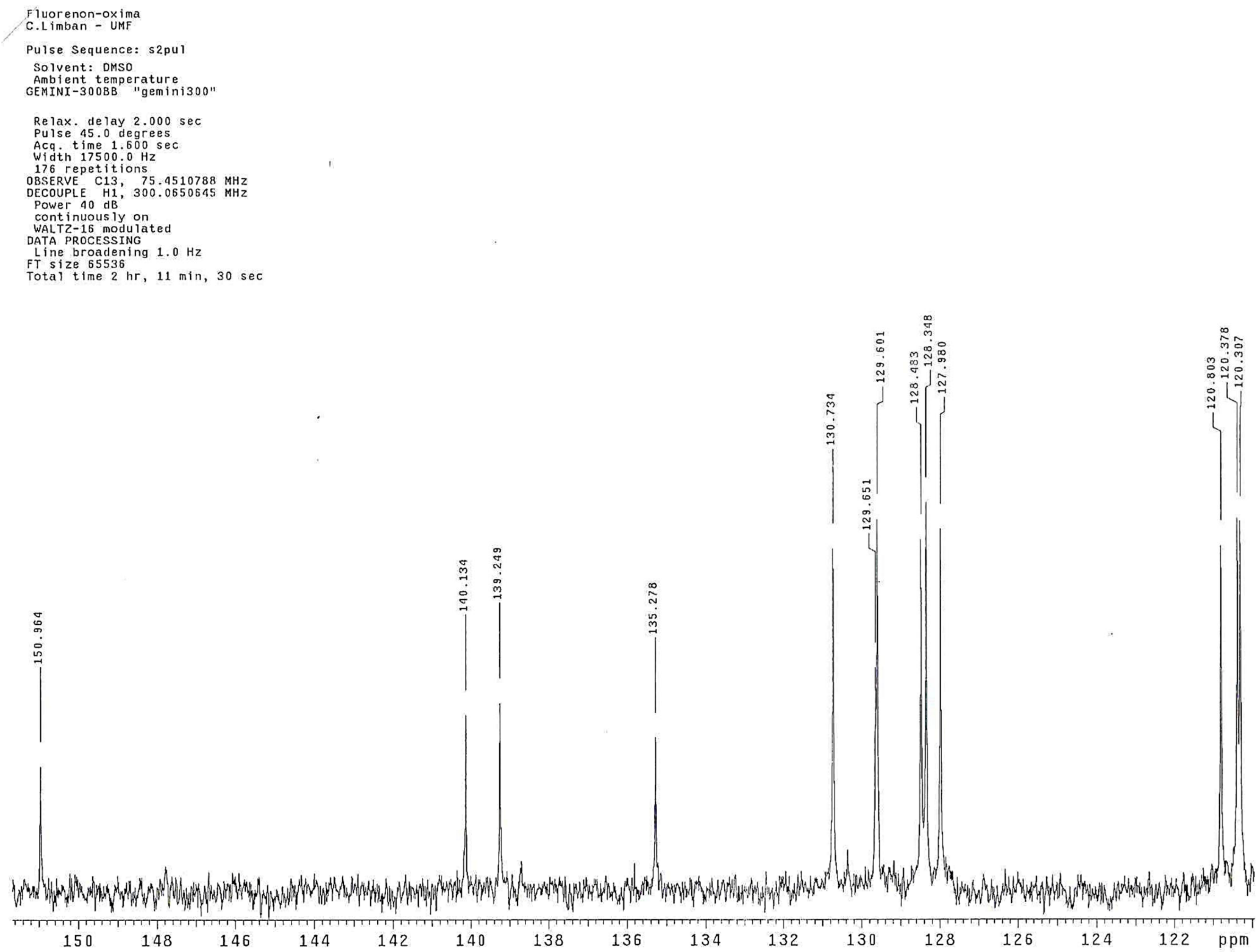

Figure S2. The ^{13}C- NMR spectra of the 9H-Fluoren-9-one Oxime.

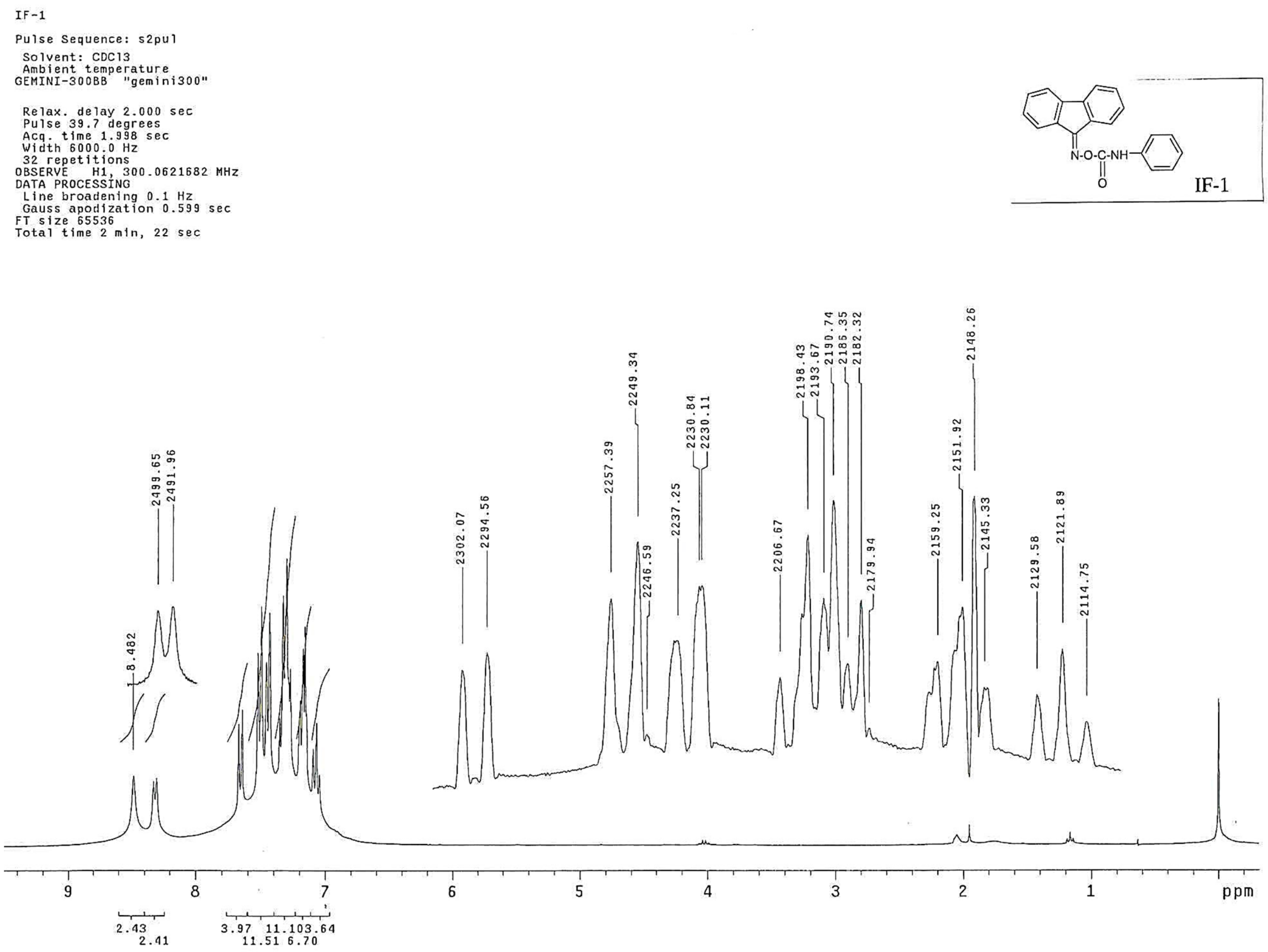

Figure S3. The ^{1}H- NMR spectra of the 9-(Phenylcarbamoyloxymino)fluorene (1a).

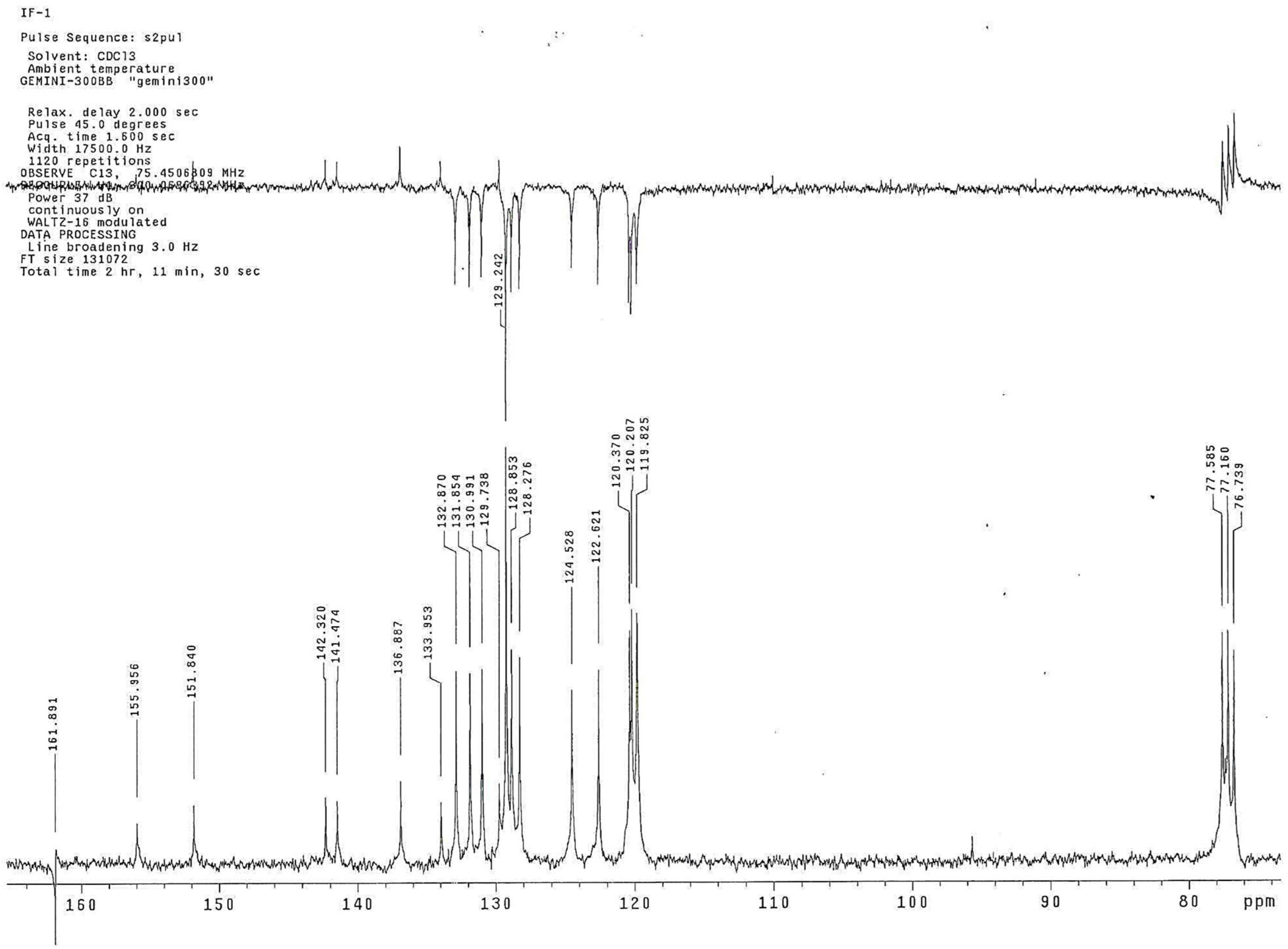

Figure S4. The ^{13}C- NMR spectra of the 9-(Phenylcarbamoyloxymino)fluorene (1a).

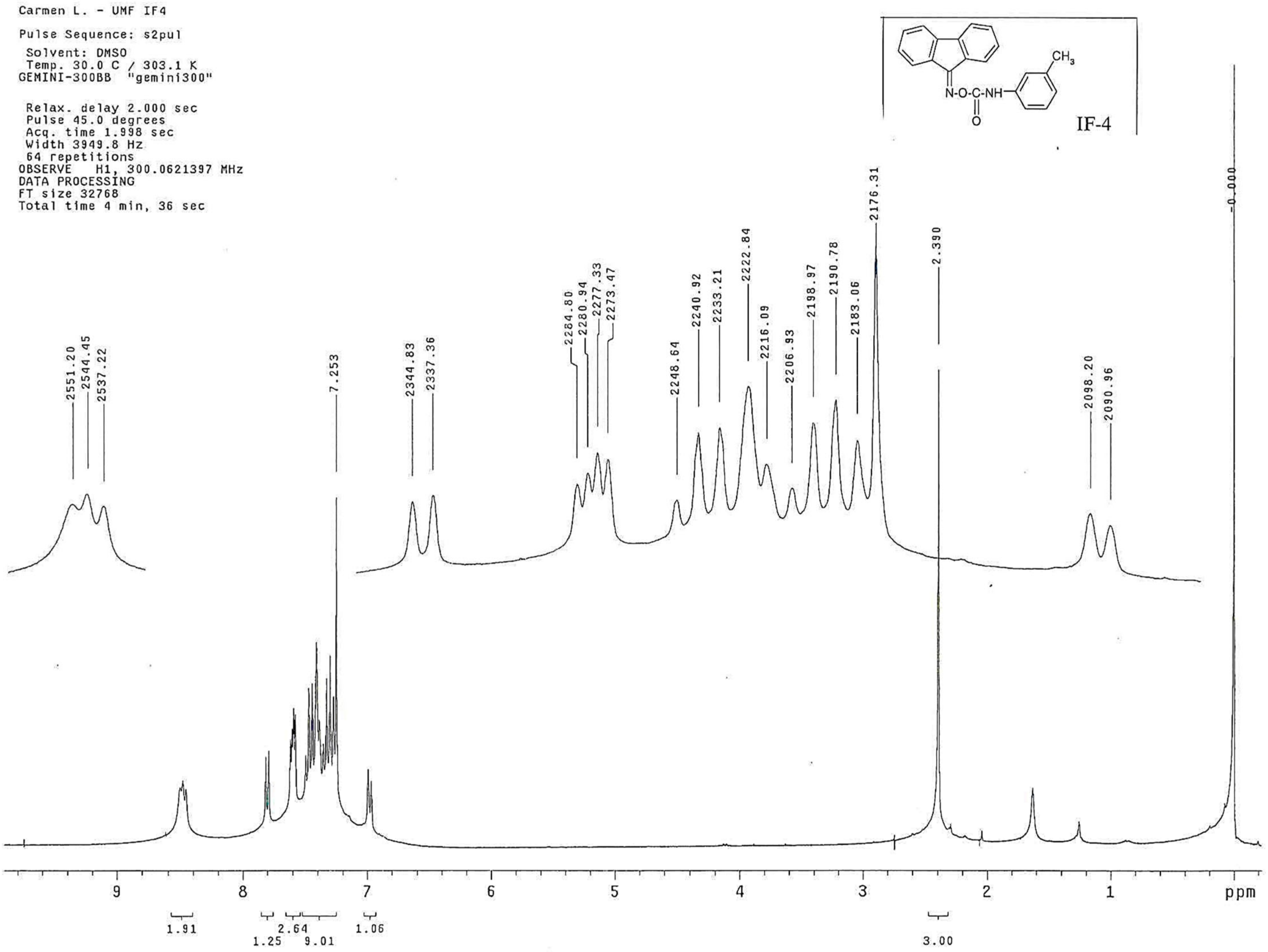

Figure S5. The ^{1}H- NMR spectra of the 9-((3-Methyl-phenyl)carbamoyloxymino)fluorene (1b).

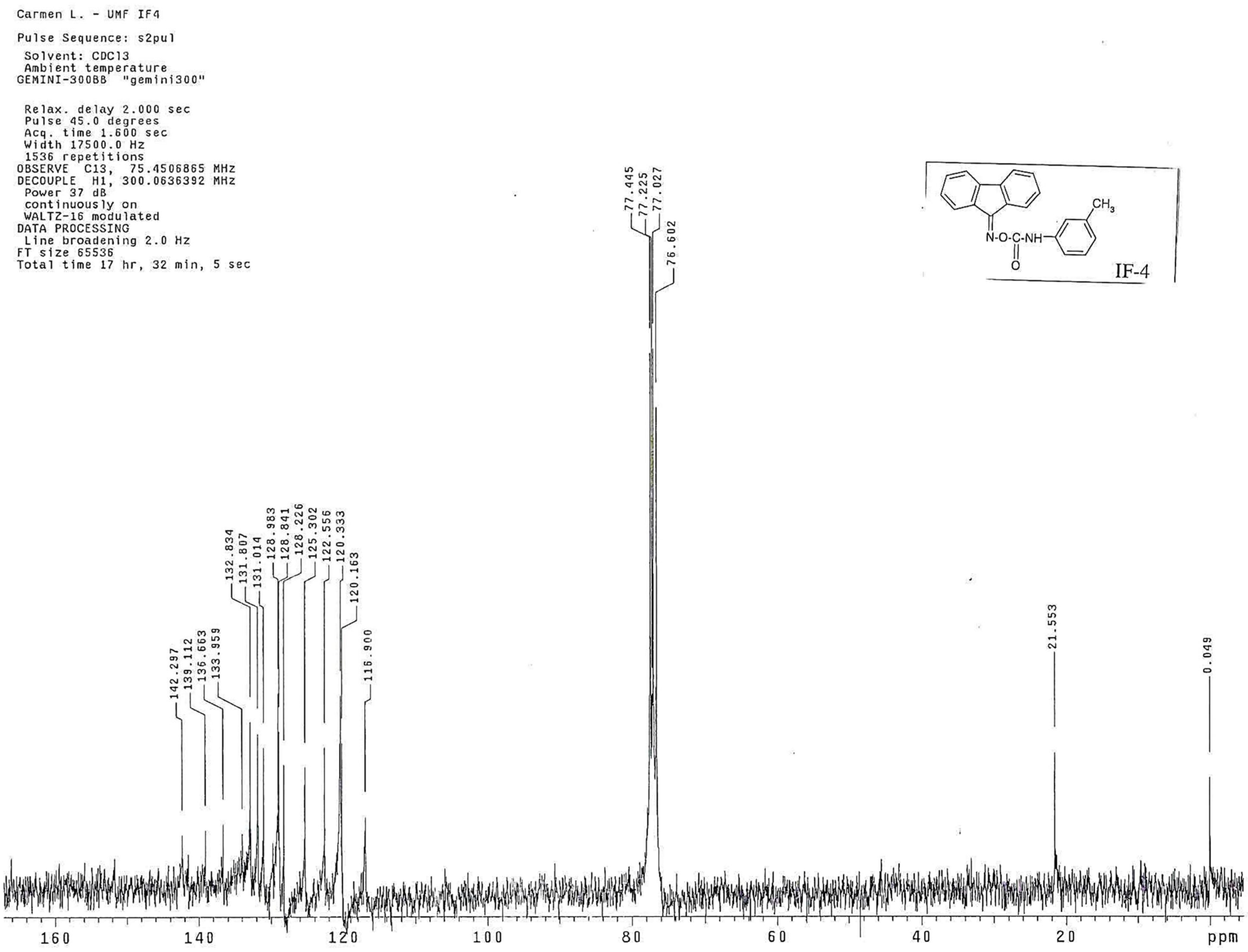

Figure S6. The ^{13}C- NMR spectra of the 9-((3-Methyl-phenyl)carbamoyloxymino)fluorene (1b)

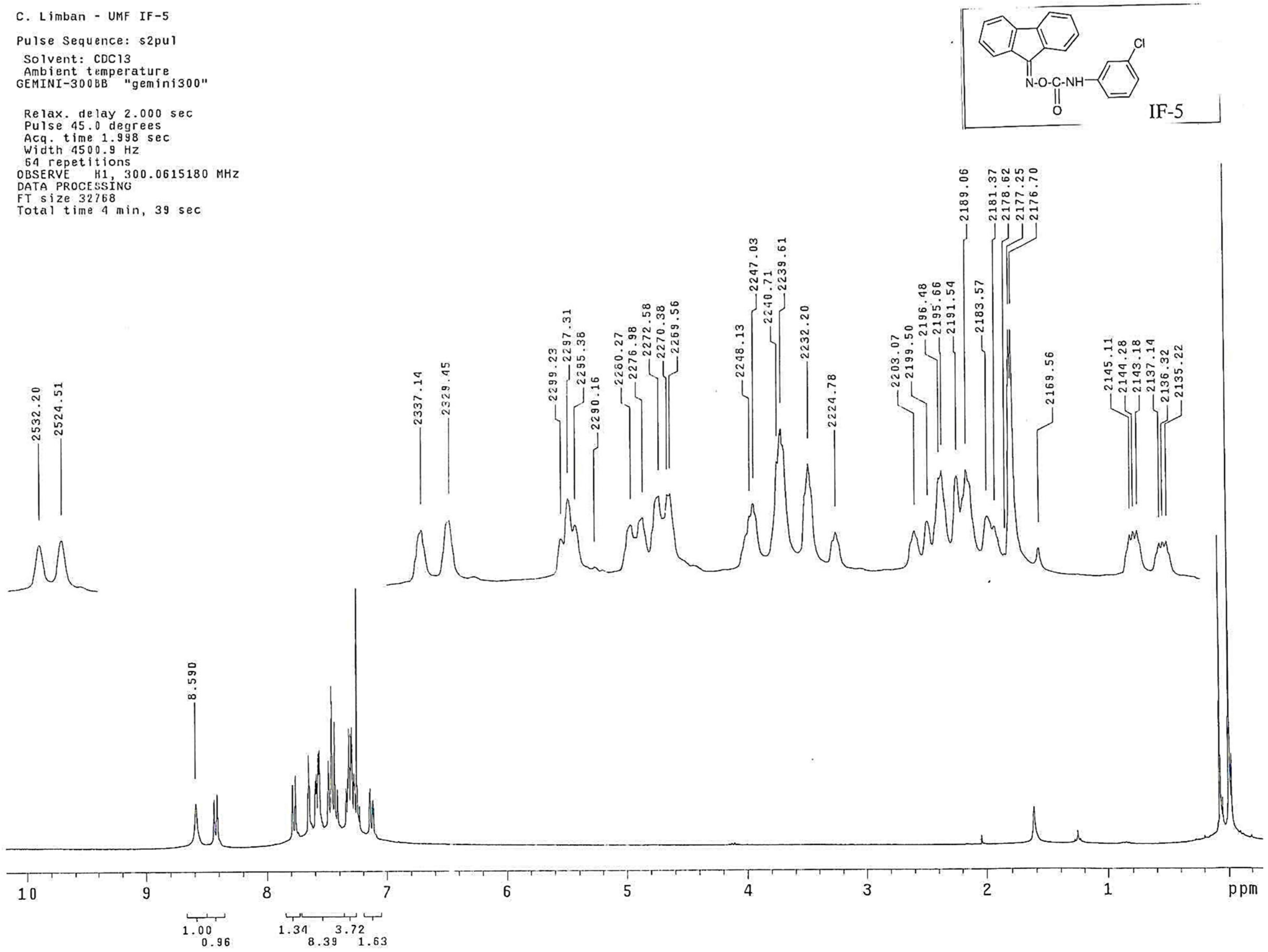

Figure S7. The ^{1}H- NMR spectra of the -((3-Chloro-phenyl)carbamoyloxymino)fluorene (1c)

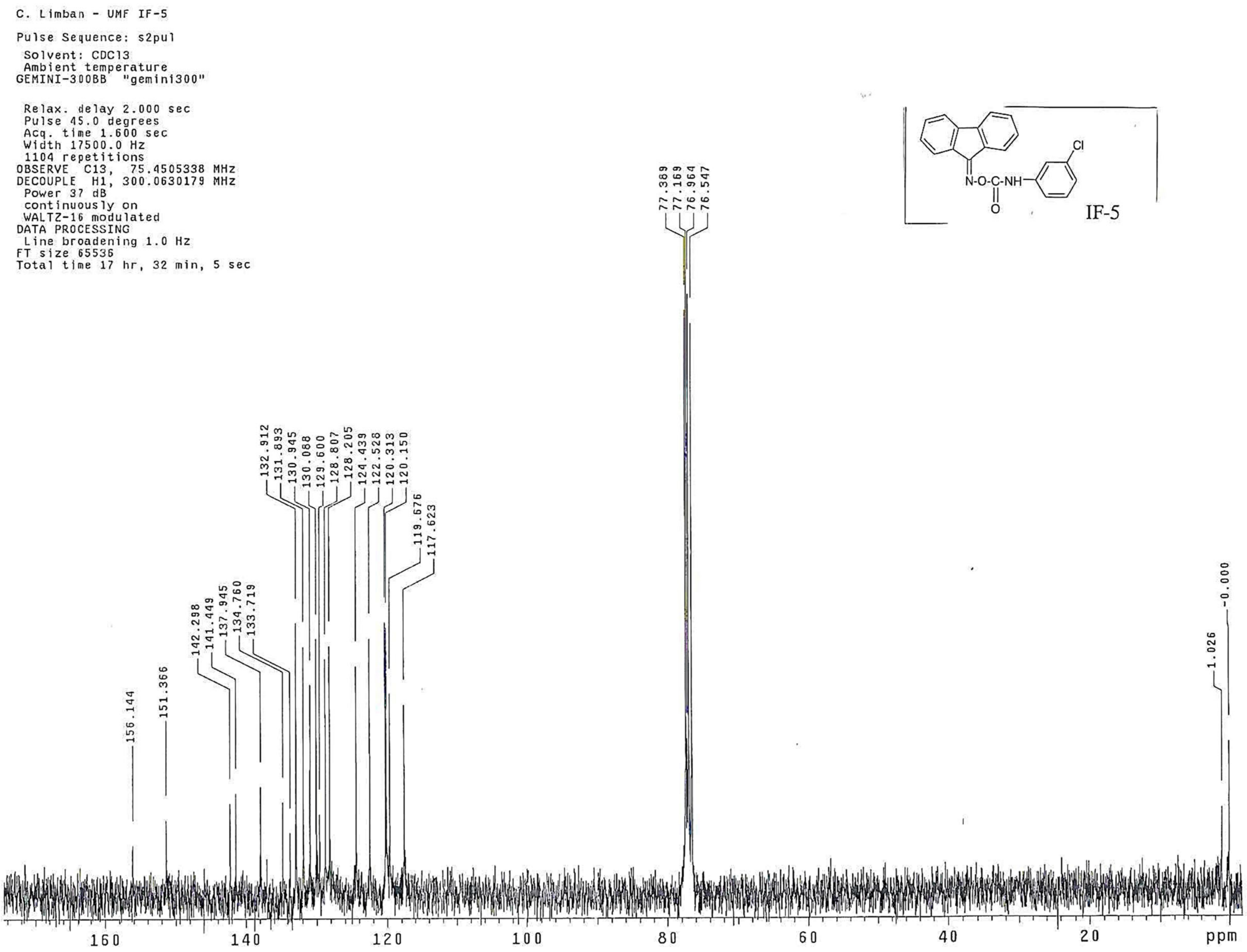

Figure S8. The ^{13}C- NMR spectra of the -((3-Chloro-phenyl)carbamoyloxymino)fluorene (1c)

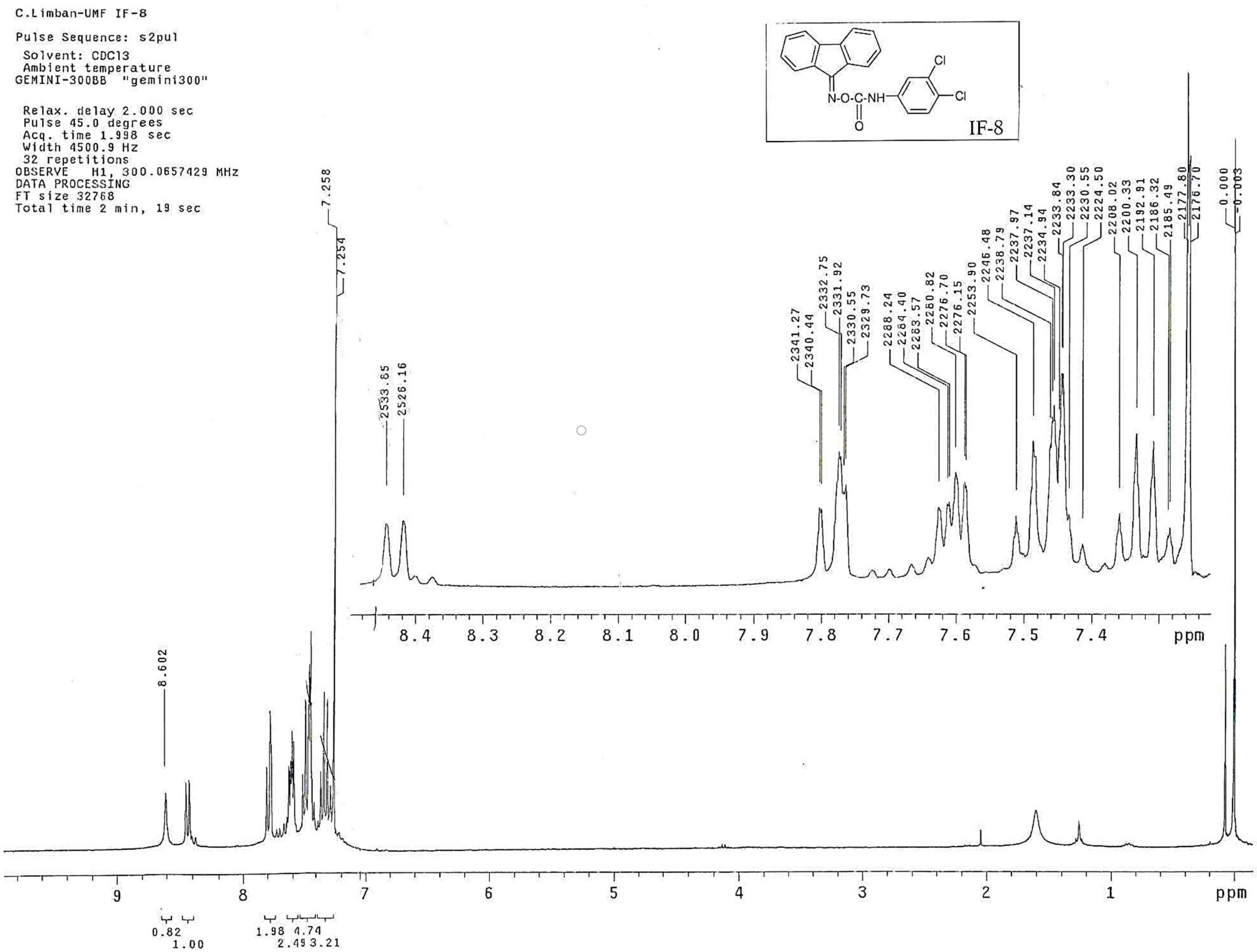

Figure S9. The ^{1}H- NMR spectra of the 9-((3,4-Dichloro-phenyl)carbamoyloxymino) fluorene (1d)

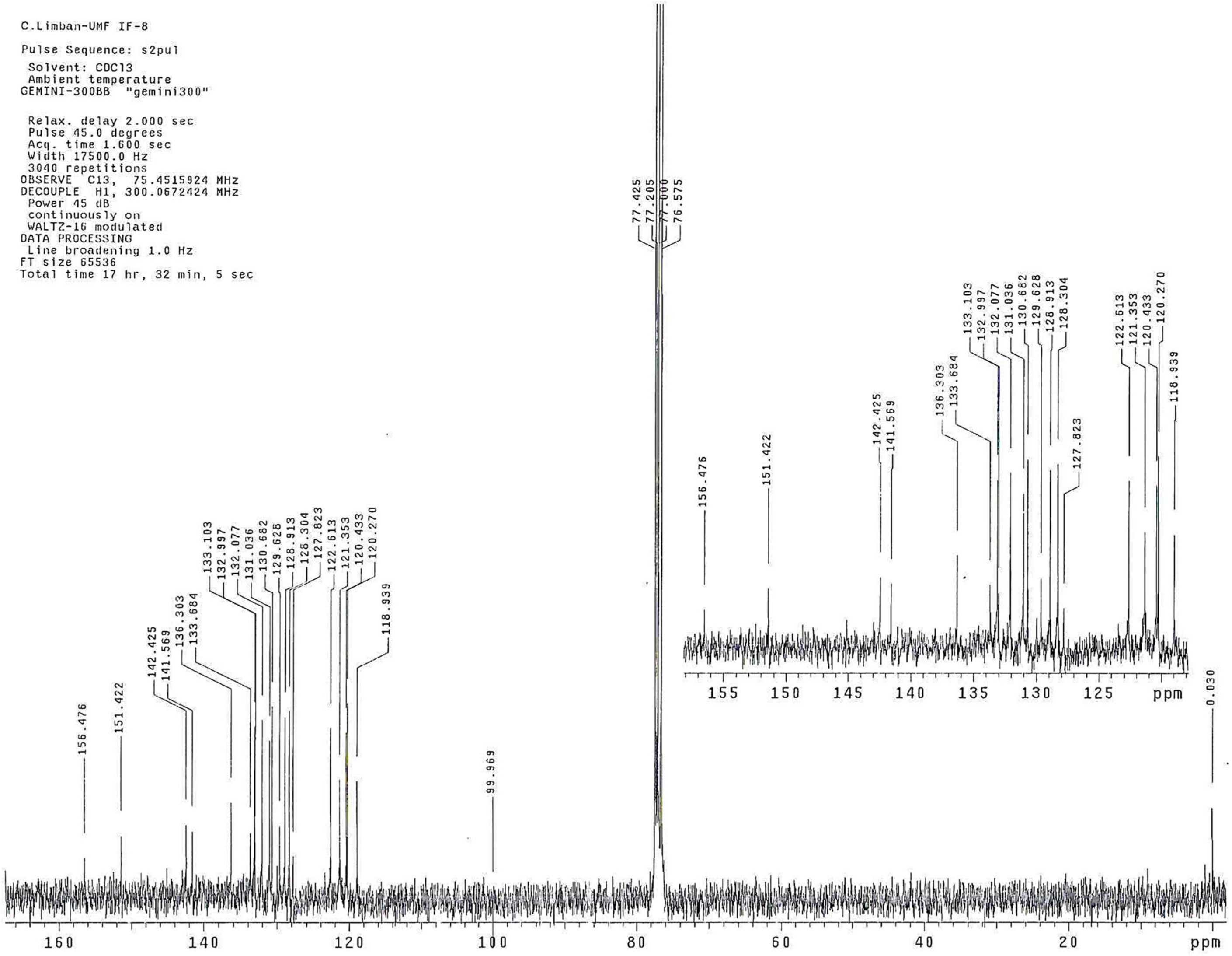

Figure S10. The ^{13}C- NMR spectra of the 9-((3,4-Dichloro-phenyl)carbamoyloxymino) fluorene (1d)
